# Serum Anti‐Erythropoietin Autoantibodies and Their Association With Younger Age in Paediatric Malaria Cases

**DOI:** 10.1155/bmri/8845434

**Published:** 2025-10-04

**Authors:** Francis Agyei Amponsah, Otchere Addai-Mensah, Lilian Antwi-Boateng, Benedict Sackey, Richard Vikpebah Duneeh, Isaac Acheampong, Prince Adoba, Diana Venunye Ama Awi, Edward Yaw Afriyie, Richard Boateng, Abrafi Ayerakwa Anokye, Veronica Agyemang, Samuel Kofi Doe, Samuel Kwasi Appiah

**Affiliations:** ^1^ Department of Medical Diagnostics, Faculty of Allied Health Sciences, Kwame Nkrumah University of Science and Technology, Kumasi, Ghana, knust.edu.gh; ^2^ Department of Medical Laboratory Sciences, University of Health and Allied Sciences, Ho, Ghana, uhas.edu.gh; ^3^ Health Information Department, Okaikwei South Sub-Metro Health Directorate, Ghana Health Service, Accra, Ghana, ghanahealthservice.org; ^4^ Department of Haematology, Komfo Anokye Teaching Hospital, Kumasi, Ghana, kathhsp.org; ^5^ Department of Serology, Komfo Anokye Teaching Hospital, Kumasi, Ghana, kathhsp.org; ^6^ Department of Biostatistics, St. John of God Hospital, Duayaw Nkwanta, Ghana; ^7^ Laboratory Department, Kintampo Health Research Center, Kintampo, Ghana; ^8^ Laboratory Department, St. John of God Hospital, Duayaw Nkwanta, Ghana; ^9^ Department of Haematology, School of Allied Health Sciences, University for Development Studies, Tamale, Ghana, uds.edu.gh

## Abstract

**Background:**

Malaria remains a major public health concern, particularly among children under 5 years in the WHO African Region. Malarial anaemia is a common complication in this population. Factors that are associated with the development of malarial anaemia include haemolysis, dyserythropoiesis, erythrophagocytosis and bone marrow suppression, with studies reporting varying erythropoietin (epo) responses to severe anaemia. Studies on anti‐epo antibodies being linked to malarial anaemia have yielded conflicting results, associated with malarial anaemia in pregnant women but not in children. This study sought to investigate anti‐epo antibody production in children with malaria and explore their association with malarial anaemia.

**Methodology:**

The study recruited 90 children aged 1–10 years in Tano North Municipality, Ghana. Of these, 60 children diagnosed with malaria (30 with anaemia and 30 without anaemia) formed the case group, while 30 healthy children served as the control group. Venous blood samples were collected into K_2_EDTA (for full blood count, G6PD activity and malaria microscopy) and serum‐separator tubes (SSTs) (sera for measurement of epo concentrations and anti‐epo antibodies using ELISA kits).

**Results:**

In all, anti‐epo antibodies were detected in 5.6% of participants who had malaria, with none of the controls being positive for the antibodies. However, the difference in anti‐epo antibody positivity between the two groups was not statistically significant. Within the subgroup of 30 malarial anaemia patients, 5.0% had anti‐epo antibodies compared to 3.37% within the subgroup of malaria without anaemia (*p* = 0.640). Antibody positivity was significantly associated with elevated epo concentrations and younger age when compared to those with malaria who did not produce anti‐epo antibodies.

**Conclusion:**

Anti‐epo antibody production is not linked to *Plasmodium falciparum* infection or malarial anaemia but is strongly associated with younger age and elevated epo levels in children.

## 1. Introduction

Malaria is a major public health concern, particularly in children under the age of 5 years in the World Health Organization’s African Region. In 2022, children under 5 years accounted for 453,005 malaria deaths out of a global total of 608,000 malaria deaths [1]. The commonest malaria complication in this population is malarial anaemia [2–4]. Management of anaemia involves the use of blood transfusion, but this is associated with the risk of disease transmission, alloimmunisation and blood scarcity [5]. Thus, the need to explore other therapeutic measures to avert and treat the anaemia is much desired. In order to find these therapeutic targets, it is equally important to know the cause of malarial anaemia.

Malarial anaemia arises from a complex interaction of factors such as haemolysis, dyserythropoiesis, erythrophagocytosis and bone marrow suppression [5–7], resulting in a significant reduction in haemoglobin (Hb) levels. However, the level of parasitaemia does not correlate with the degree of anaemia, as observed in one study, but for each parasitised erythrocyte destroyed, eight nonparasitised erythrocytes are simultaneously destroyed [8]. The body’s response to the ensuing anaemia is the production and release of erythropoietin (epo) from the kidneys for the production of erythrocytes by the bone marrow. In malarial anaemia, the level of epo response has been conflicting, with some studies reporting inadequate [9, 10], adequate [11] and elevated [12] epo levels for the degree of anaemia. Nevertheless, the epo response in most cases is unable to avert the progression of the anaemia to severe anaemia, which brings to fore the possibility of a serum factor that inhibits epo’s activity.

Serum factors that have been found to be associated with epo resistance include anti‐epo antibody and anti‐epo receptor antibody [13]. Of the two, anti‐epo antibody has been extensively studied and found to be associated with the development of anaemia in various conditions such as pure red cell aplasia [14–17], renal anaemia [18] and human immunodeficiency viral‐1 (HIV‐1) infections [19]. Apart from the aforementioned conditions, anti‐epo antibody is responsible for anaemia in individuals undergoing treatment with erythropoiesis stimulating agents (ESAs) [20]. Research on the antibody’s production is limited in malaria. However, the earliest study to be conducted in malaria reported that the antibody is produced in murine malaria, and its production was associated with anaemia [11]. Thus, similar studies have been conducted in human malaria but with conflicting results. In our earlier study, we found that anti‐epo antibodies are not produced in human malaria and that any observed anaemia was likely due to increased haemolysis [2]. This finding was contrasted by a later study conducted in the Northern Region of Ghana, which reported that anti‐epo antibody is produced in *Plasmodium*‐positive pregnant women and associated with malarial anaemia [3]. The contradictory findings of both studies require the need to reassess the production of anti‐epo antibody especially in children with malaria. Thus, the present study sought to find out if anti‐epo antibody is produced in children with malaria and whether the antibody is associated with malarial anaemia. The success of this study will enhance our understanding of the pathophysiology underlying malarial anaemia especially in children.

## 2. Materials and Methods

### 2.1. Study Design and Study Site

This is an unpaired case‐control study in Ghanaian children with malarial anaemia at the Outpatient Department of the St. John of God Catholic Hospital, Duayaw Nkwanta, Ahafo Region. The St. John of God Catholic Hospital is the main referral hospital in the Tano North Municipal and provides services in obstetrics and gynaecology, orthopaedic and trauma, antenatal care, orthotics and physiotherapy, eye clinic, ENT (ear, nose and throat), internal medicine, pharmacy, laboratory, medical imaging and so on. Duayaw Nkwanta is located on the Kumasi–Sunyani highway. Tano North has a population of 77,000 people. It is bounded in the North‐West by Sunyani Municipality and in the South‐West by Asutifi district to the South by Tano South and to the North by Offinso districts. It has two main seasons: the rainy seasons which start from April to October and dry season (November to March).

### 2.2. Study Population and Sample Size

This is an unpaired case‐control substudy of a larger cohort study involving 244 *Plasmodium*‐positive children. In this study, the case group consisted of children aged 1–10 years who were conveniently selected from the cohort of 244 laboratory‐confirmed *Plasmodium*‐positive individuals, whereas the control group comprised apparently healthy children within the same age range. The sample size for this study was calculated with EPI INFO using the Kelsey unmatched case‐control formula. The parameters used were two‐sided confidence level = 95*%*; power = 90*%*; ratio of controls to cases = 0.5; proportion of controls with exposure, that is, prevalence of anaemia in children under 5 years = 48.9*%* [21] and proportion of cases with anaemia, that is, average prevalence of malaria in children 6 months to 10 years = 4.1*%* [22]. A total of 38 participants, comprising 25 cases and 13 controls, were obtained. However, to further increase the power of the study, 60 cases and 30 controls were used. The case group was equally divided between children with malarial anaemia and those with malaria but no anaemia.

### 2.3. Inclusion and Exclusion Criteria

Children aged 1–10 years whose parents voluntarily consented to the study were included. Children with other comorbidities such as sickle cell anaemia, helminthiasis, HIV/AIDS, hepatitis C viral infection and hepatitis B viral infection were excluded. Sickle cell anaemia was diagnosed with a sodium metabisulphite test, routine stool examination for helminthiasis and rapid diagnostic test kits for HIV, hepatitis C and hepatitis B viral infections. Controls that had been on antimalarial therapy in the last 3 months to their enrolment in the study were also excluded.

### 2.4. Ethical Consideration

Ethical approval for the study was sought from the Committee on Human Research Publications and Ethics of the School of Medical Sciences, Kwame Nkrumah University of Science and Technology (CHRPE/AP/794/23). Permission was obtained from the Hospital Management Team (HMT) of the St. John of God Catholic Hospital prior to participants’ recruitment. Written informed consent was also obtained from participants’ parents or guardians before their recruitment.

### 2.5. Sample Collection and Processing

Venous blood samples were aseptically taken into dipotassium ethylenediaminetetraacetic acid (K_2_EDTA) tubes (3 mL) and serum‐separator tubes (3 mL) before participants were put on antimalarial therapy. K_2_EDTA anticoagulated blood samples were used for full blood count (FBC), glucose‐6‐phosphate dehydrogenase (G6PD) activity and malaria microscopy (thick and thin blood films), while sera obtained from the SST blood samples were frozen at −20°C for subsequent assaying of epo and anti‐epo antibodies using human epo enzyme‐linked immunosorbent assay (ELISA) kits and human anti‐epo antibody ELISA kit (Jiuqiang Biotechnology Co. Ltd, Beijing, China), respectively, in accordance with the manufacturer’s instructions. Participants’ anti‐epo antibody positivity or negativity was determined by the method used by Addai‐Mensah et al. [2].

FBC test was carried out using the Sysmex XN‐350 fully automated compact six‐part differential haematology analyser (Sysmex Corporation, Japan). This analyser incorporates fluorescence flow cytometry, hydrodynamic focusing and cyanide‐free SLS method for estimating Hb levels. Thick and thin blood films for malaria microscopy were stained with 10% Giemsa solution for 10 min for malaria parasite identification, speciation and count. Malaria was defined as the presence of asexual forms of *Plasmodium* species on microscopy and ill health. Blood films were reported as negative when no parasites were found after examining 200 oil‐immersion fields. Parasite density was calculated using the following formula: number of parasites counted/WBC counted × WBC count/*μ*L of participant^’^s blood.

Malarial anaemia was defined as the presence of asexual forms of *Plasmodium* species and Hb concentration of less than 11.0 g/dL for children aged 1–10 years [2].

G6PD activity was measured on EDTA anticoagulated venous blood samples with the G6PD assay kit (Beijing Strong Biotechnologies Inc., P.R. China) in accordance with the manufacturer’s protocol. To account for interindividual differences in erythrocyte size and concentration, enzyme activity was normalised for Hb levels and expressed as G6PD/Hb.

### 2.6. Statistical Analysis

The study’s data were entered into Microsoft Excel and later analysed using International Business Machines Corporation’s Statistical Package for the Social Sciences (IBM SPSS) software Version 26.0 (IBM Corp., Armonk, NY, United States). Categorical variables were presented as frequencies (percentages), and tests for association were performed using the chi‐square test statistic or Fisher’s exact where appropriate. The Shapiro–Wilk and Kolmogorov–Smirnov normality tests were conducted for continuous data. Nonparametric data were presented as medians (25^th^–75^th^ percentiles), and the Mann–Whitney nonparametric method was used to compare data between participants in each group. Box plots were used to illustrate the comparison of haematological parameters and parasite density among the participants. The Kruskal–Wallis test with multiple comparison was used to compare anti‐epo antibody levels among participants with malaria only, both malaria and anaemia and the healthy controls. The Mann–Whitney test was used to compare haematological parameters and parasite densities between malaria cases with and without anti‐epo antibodies. *p* < 0.05 was considered statistically significant for all comparisons.

## 3. Results

### 3.1. Baseline Characteristics of Study Participants

A total of 90 children aged 1–10 years from the Tano North Municipal were recruited for this case‐control study. Sixty out of the 90 children were diagnosed with malaria, and this served as the case group, while the remaining 30 healthy children constituted the control group. The majority of the study’s participants (54.4%) were males, and the same was observed across both groups. The median age for the entire study population was 5 (3–8) years with cases being significantly younger (*p* value = 0.013) than controls. Most study participants sleep under insecticide‐treated nets (ITNs), a pattern similar in both cases and controls (*p* > 0.05). The case group presented with higher body temperature than control (37.5°C vs. 36.9°C, *p* = 0.001). FBC analysis revealed significantly higher neutrophils count (4.3 × 10^9^/L vs. 3.4 × 10^9^/L, *p* = 0.024) than the controls but a lower haematocrit (34.4% vs. 35.4%, *p* = 0.023), RDW‐CV (12.2% vs. 13.1%, *p* = 0.001), lymphocyte count (1.3 × 10^9^/L vs. 2.1 × 10^9^/L, *p* = 0.001), eosinophil count (0.0 × 10^9^/L vs. 0.2 × 10^9^/L, *p* = 0.001), basophil count (0.01 × 10^9^/L vs. 0.02 × 10^9^/L, *p* = 0.001), platelet count (131.0 × 10^9^/L vs. 241.0 × 10^9^/L, *p* = 0.001) and MPV (10.0 vs. 10.5, *p* = 0.004). Also, case and control groups had similar Hb concentration (11.2 g/dL vs. 11.7 g/dL, *p* = 0.066) and serum epo concentration (3.2 IU/L vs. 4.5 IU/L, *p* = 0.162) (Table 1).

**Table 1:** The demographics and clinicohaematological characteristics of the study’s participants.

	**Total number of participants:** **N** = 90	**Malaria cases: 60 (66.7%)**	**Healthy controls: 30 (33.3%)**	**p** **value**
Age (years) (median, IQR)	5 (3–8)	4 (3–7)	7 (4–9)	**0.013**
Gender, *N* (%)				0.881
Male	49 (54.4%)	33 (36.7%)	16 (17.8%)	
Females	41 (45.6%)	27 (30.0%)	14 (15.6%)	
Use of insecticide‐treated nets (ITNs)				1.00
Yes	60 (66.7%)	40 (44.4%)	20 (22.2%)	
No	30 (33.3%)	20 (22.2%)	10 (11.1%)	
Weight (kg) (median, IQR)	18.4 (14.0–25.7)	16.5 (13.4–21.5)	24.0 (15.0–28.0)	0.051
Temperature (°C) (median, IQR)	37.2 (36.8–37.8)	37.5 (36.9–38.4)	36.9 (36.8–37.1)	**0.001**
RBC count (×10^9^/L) (median, IQR)	4.5 (4.1–4.8)	4.5 (3.8–5.0)	4.5 (4.3–4.7)	0.524
Haemoglobin concentration (g/dL)	11.6 (10.4–12.4)	11.2 (9.5–12.6)	11.7 (11.4–12.4)	0.066
Haematocrit (%)	34.9 (30.3–37.1)	34.4 (28.5–36.7)	35.4 (34.8–37.1)	**0.023**
MCV (fL) (median, IQR)	75.4 (71.4–79.8)	75.0 (71.3–79.5)	75.8 (72.1–80.5)	0.620
RDW‐CV (%) (median, IQR)	12.7 (11.8–13.7)	12.2 (11.3–13.4)	13.1 (12.8–14.1)	**0.001**
WBC count (median, IQR)	6.2 (4.9–8.3)	6.2 (4.3–9.0)	6.3 (5.8–6.8)	0.827
Neutrophil count (×10^9^/L) (median, IQR)	3.6 (2.9–5.7)	4.3 (2.9–6.5)	3.4 (2.9–4.0)	**0.024**
Lymphocyte count (×10^9^/L) (median, IQR)	1.6 (1.1–2.2)	1.3 (0.9–1.9)	2.1 (1.6–2.6)	**0.001**
Monocyte count (×10^9^/L) (median, IQR)	0.4 (0.3–0.6)	0.4 (0.2–0.6)	0.5 (0.4–0.6)	0.059
Eosinophil count (×10^9^/L) (median, IQR)	0.1 (0.0–0.2)	0.0 (0.0–0.1)	0.2 (0.1–0.4)	**0.001**
Basophil count (×10^9^/L) (median, IQR)	0.0 (0.0–0.0)	0.01 (0.00–0.01)	0.02 (0.02–0.03)	**0.001**
Platelet count (×10^9^/L) (median, IQR)	187.0 (110.0–260.0)	131.0 (82.0–210.5)	241.0 (195.0–309.0)	**0.001**
MPV	10.1 (9.7–10.6)	10.0 (9.6–10.3)	10.5 (10.1–11.0)	**0.004**
Epo concentration (IU/L)	3.6 (0.5–7.2)	3.2 (0.2–5.9)	4.5 (1.8–7.8)	0.162
Anti‐epo antibody				0.104
Positive	5 (5.6%)	5 (5.6%)	0 (0.0%)	
Negative	85 (94.4%)	55 (61.1%)	30 (33.3%)	

*Note:* The median age of children with malaria was less than 5 years, and no statistically significant difference was found in epo concentration compared between both groups. Mann–Whitney test was used to test differences between continuous variables of cases and controls. Chi‐square and Fisher’s exact test were used to test for association between categorical variables. A *p* value < 0.05 was deemed statistically significant.

Abbreviations: CV, coefficient of variation; epo, erythropoietin; IQR, interquartile range; ITN, insecticide‐treated net; MCV, mean cell volume; MPV, mean platelet volume; N, number of participants; RBC, red blood cell; RDW, red cell distribution width; WBC, white blood cell.

### 3.2. Relationship Between Anti‐Epo Antibody and Some Haematological Parameters, Parasite Density, Epo Levels and Age

**Figure 1:**
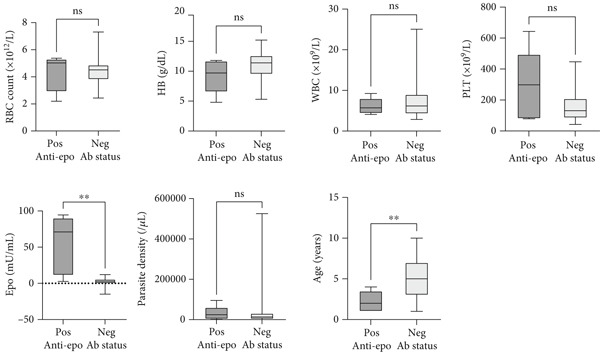
Comparison of age, full blood count parameters, erythropoietin (epo) and parasite density between malaria cases with and without EPO antibodies in children (Mann–Whitney *U* test was used for all comparisons). Pos, positive; Neg, negative; epo, erythropoietin levels; anti‐epo ab, antierythropoietin antibodies; RBC, red blood cells; Hb, haemoglobin concentration; WBC, white blood cells; PLT, platelet counts; ns, not statistically significant.  ^**^Statistically significant.

The median serum epo levels were significantly higher (*p* = 0.001) among *Plasmodium falciparum* infected children with positive anti‐epo antibodies than those with negative anti‐epo antibodies. Also, the average age of *P. falciparum* infected children with positive anti‐epo antibodies was significantly lower (*p* = 0.006) than those negative for anti‐epo antibodies. However, RBC count, Hb concentration, platelet count, WBC count and parasite density were similar (*p* > 0.05) between *P. falciparum*–infected children with positive serum anti‐epo antibodies and those with negative serum anti‐epo antibodies (Figure 1).

### 3.3. Demographics and Clinicohaematological Characteristics of Children With Malaria Stratified by Anaemia

To better understand the cause of the malarial anaemia, the case group was divided into two groups: those with malarial anaemia and those with malaria without anaemia, as seen in Table 2. Younger age was significantly associated with malarial anaemia compared to malaria without anaemia (4.0 years [2.0–5.0] vs. 6.5 years [4.0–8.0], *p* = 0.008). Similarly, those with malarial anaemia had lower weight (15.0 years [12.1–19.4] vs. 19.5 years [14.4–26.0], *p* = 0.032), RBC count (4.0 × 10^9^/L [3.1–4.4] vs. 4.9 × 10^9^/L [4.5–5.3], *p* = 0.001), Hb concentration (9.5 g/dL [7.9–10.4] vs. 12.6 g/dL [11.9–13.2], *p* = 0.001), haematocrit (28.5% [25.0–30.3] vs. 36.6% [34.8–39.5], *p* = 0.001), MCV (73.5 fL [69.1–6.7] vs. 78.6 fL [73.7–81.5], *p* = 0.013) and MPV (9.9 [9.5–10.1] vs. 10.1 [9.7–10.6], *p* = 0.040) compared to those with malaria without anaemia. On the other hand, WBC count, neutrophil count, RDW‐CV, platelet count and epo concentration did not show significant variations between children with malaria anaemia compared to those with malaria without anaemia. Of the 90 children recruited for the study, anti‐epo antibodies were detected in 5.6% of them. Among the 60 children who had malaria, 8.3% had anti‐epo antibodies in their serum, and within the subgroup of 30 malarial anaemia patients, 5.0% had anti‐epo antibodies compared to 3.37% within the subgroup of malaria without anaemia, but the difference was not statistically significant (*p* = 0.640).

**Table 2:** The demographics and haematological characteristics of the case group stratified by malarial anaemia.

	**Malarial anaemia: 30 (50.0%)**	**Malaria nonanaemic: 30 (50.0%)**	**p** **value**
Age (years), median (IQR)	4.0 (2.0–5.0)	6.5 (4.0–8.0)	**0.008**
Gender, *N* (%)			0.299
Male	19 (31.7%)	14 (23.3%)	
Females	11 (18.3%)	16 (26.7%)	
Weight (kg), median (IQR)	15.0 (12.1–19.4)	19.5 (14.4–26.0)	**0.032**
Temperature (°C), median (IQR)	37.5 (36.9–38.4)	37.5 (36.8–38.4)	0.773
RBC count (×10^9^/L), median (IQR)	4.0 (3.1–4.4)	4.9 (4.5–5.3)	**0.001**
Haemoglobin concentration (g/dL), median (IQR)	9.5 (7.9–10.4)	12.6 (11.9–13.2)	**0.001**
Haematocrit (%)	28.5 (25.0–30.3)	36.6 (34.8–39.5)	**0.001**
MCV (fL), median (IQR)	73.5 (69.1–76.7)	78.6 (73.7–81.5)	**0.013**
RDW‐CV (%), median (IQR)	11.9 (11.2–13.6)	12.3 (11.3–13.1)	0.625
WBC count (×10^9^/L), median (IQR)	6.0 (4.2–8.2)	6.9 (4.5–9.7)	0.204
Neutrophil count (×10^9^/L), median (IQR)	3.8 (2.8–6.2)	4.3 (3.3–7.2)	0.212
Lymphocyte count (×10^9^/L), median (IQR)	1.4 (1.0–1.9)	1.2 (0.7–2.0)	0.333
Monocyte count (×10^9^/L), median (IQR)	0.4 (0.3–0.6)	0.4 (0.2–0.6)	0.762
Eosinophil count (×10^9^/L), median (IQR)	0.0 (0.0–0.1)	0.0 (0.0–0.1)	0.552
Basophil count (×10^9^/L), median (IQR)	0.0 (0.0–0.0)	0.0 (0.0–0.1)	0.240
Platelet count (×10^9^/L), median (IQR)	103.5 (79.0–162.0)	160.0 (113.0–228.0)	0.510
MPV	9.9 (9.5–10.1)	10.1 (9.7–10.6)	**0.040**
Epo (IU/L)	2.4 (0.1–4.9)	3.5 (0.2–7.2)	0.510
Anti‐epo antibody			0.640
Positive	3 (5.0%)	2 (3.3%)	
Negative	27 (45.0%)	28 (46.7%)	
Parasite density (mps/*μ*L of blood)	15077 (5075–30581)	9746 (2146–30736)	0.433
G6PD activity (U/L), median (IQR)	1580.5 (1053.0–1885.0)	1885.0 (1774.0–2162.0)	**0.001**
G6PD/Hb (U/gHb), median (IQR)	15.9 (13.1–19.1)	16.2 (13.2–17.7)	0.712

*Note:* Malarial anaemia is very common among children under 5 years, and although parasite density was very high in those with malarial anaemia, the difference was not statistically significant (*p* = 0.433) compared to those with malaria, but no anaemia.

Abbreviations: CV, coefficient of variation; epo, erythropoietin; G6PD, glucose‐6‐phosphate dehydrogenase; G6PD/Hb, standard unit of G6PD activity; IQR, interquartile range; MCV, mean cell volume; MPV, mean platelet volume; N, number of participants, RBC, red blood cell; RDW, red cell distribution width; WBC, white blood cell.

Children with malarial anaemia had significantly lower G6PD activity compared to those with malaria without anaemia (1580.5 U/L [1053.0–1885.0] vs. 1885.0 U/L [1774.0–2162.0], *p* = 0.001). However, when G6PD activity was adjusted by Hb concentration to produce the standard unit of the enzyme’s activity, no significant difference was observed between malaria cases with anaemia and those without anaemia (15.9 U/gHb [13.1–19.1] vs. 16.2 U/gHb [13.2–17.7], *p* = 0.712) (Table 2).

## 4. Discussion

The aetiology of malarial anaemia has traditionally included haemolysis, dyserythropoiesis, erythrophagocytosis and bone marrow suppression [5, 7, 11], but recently, the possibility of autoimmunity as a cause of malarial anaemia is gaining attention. This stems from the discovery of anti‐epo antibody involvement in anaemia in diseases such as systemic lupus erythromatosus, HIV/AIDS and murine malaria studies [11, 14, 15, 17–19, 23]. Children under 5 years are the most vulnerable group to malaria infection, and the disparity of *Plasmodium* parasitaemia level and severity of anaemia is a regular unexplained finding. This case‐control study seeks to decipher the association between anti‐epo antibodies and human malarial anaemia in children.

It was observed that malaria infection was more prevalent in younger children than older children despite a comparable use of insecticide‐treated bed nets, confirming similar findings reported in other studies [1, 2]. Individuals living in malaria endemic regions may acquire a certain level of antimalarial immunity after repeated exposure to the parasite or premunition. It is reported that anti‐merozoite surface antigen 1 (anti‐MSP‐1) formed against P. *falciparum*, for instance, was associated with afebrile malaria with less parasitaemia and loss of Hb. The recognition of higher protective levels of antimalaria antibodies forming among older age children with more exposure may explain the unique risk of younger age children to higher parasitemia, febrile malaria and other worse clinical outcomes [2, 24, 25]. This malaria‐associated fever is believed to be due to toll‐like receptor 9 (TLR9) stimulation by plasmodia DNA, and this helps control the infection by killing the parasite [25].

This study found no significant differences in RBC count, Hb concentration, WBC counts and epo levels between children infected with *P. falciparum* malaria and healthy uninfected children, but children diagnosed for malaria had a significantly lower haematocrit, which supports previous findings that Hb and haematocrit may not correlate reliably in malaria cases [26, 27]. In agreement with other studies [28–30], individuals with malaria had significantly lower platelet counts compared to the healthy controls. Platelets are known to either confer protection against malaria by directly killing parasitized erythrocytes or by promoting the disease’s severity via facilitation of cytoadherence of parasitized erythrocytes, and these processes could account for the observed decreased thrombocyte counts in children diagnosed for malaria [29, 30].

Of the 90 study participants recruited, anti‐epo antibodies were detected in 5.6% of them (Table 1). The result is similar to that reported among pregnant women with *P. falciparum* infection in northern Ghana [3]. Contrarily, this study did not observe any association between anti‐epo antibodies and malarial anaemia as was observed in *P. falciparum* infection in Ghanaian pregnant women. The observed differences could be attributed to differences in study designs and study population: children between 1 and10 years for the present study while pregnant women were recruited for the other study. Although in the present study, *P. falciparum* malaria was not associated with anti‐epo antibody production which agrees with our previous study [2], the two studies differ in terms of the prevalence of anti‐epo antibodies in their respective control groups (0% vs, 1.2% for the present study and previous study, respectively) and for the entire study populations (5.6% for the present study against 3.5% for the previous study). The observed disparity in anti‐epo antibody prevalence in the control groups of both studies could be attributed to the fact that controls for the present study were recruited if they had not been treated for malaria for at least 3 months while for the previous study, the duration was just a month prior to recruitment. Thus, the positive antibody in the control participant could arise from a very recent but treated *Plasmodium* infection.

Contrary to other research findings [3], the presence of anti‐epo antibody in children with *P. falciparum* malaria did not significantly affect Hb level, parasite count, WBC count and platelet count (Figure 1). However, it was observed that *P. falciparum* infected children who tested positive for anti‐epo antibody were significantly of younger age and had significantly higher serum epo levels compared to *P. falciparum* infected children who tested negative for serum anti‐epo antibody. This result confirms similar reports by Nkansah et al. and Schett et al. [3, 23], respectively. The very high epo levels in children who tested positive for anti‐epo antibodies suggest that these antibodies could be neutralising epo’s biological activity. As a result, the kidneys continue to produce excess epo in response to ongoing tissue hypoxia. The association between anti‐epo antibodies and younger age, however, is not fully understood and thus the need for more research in that regard. The higher epo levels associated with younger age, however, protect younger children living in malaria endemic regions from cerebral malaria but not malarial anaemia and thus explain why malaria anaemia is very common in younger children [6, 30].

It was also observed that children with malarial anaemia were significantly younger than those with malaria but no anaemia. This finding buttresses the point that malarial anaemia is very common in children under 5 years as reported by other studies [31–34]. WBC and platelet counts were insignificantly lower in children with malarial anaemia compared to those with malaria without anaemia. More so, findings from this study support the observation that parasite count does not influence the development of malarial anaemia even though children with malarial anaemia had higher parasite density as compared to their malaria nonanaemic counterparts. It can be inferred from the significantly lower RBC count in children with malarial anaemia that their anaemia is likely due to either increased haemolysis or suppressed erythropoiesis. In a recent study in Ethiopia, participants with acute plasmodia infections had suppressed G6PD activity [35] which could account for both increased haemolysis and suppressed erythropoiesis as observed in the present study. However, a comparison of G6PD activity between children diagnosed with malarial anaemia and those with malaria without anaemia showed significantly lower enzymatic activity in *P. falciparum* infected children with anaemia compared to those without anaemia. However, adjusting G6PD activity by Hb concentration to produce the standard unit of the enzyme’s activity negated any previously observed significant differences suggesting that reduced G6PD activity may not be the cause of the observed malarial anaemia. This assumption may however be saddled in the present study with a smaller sample size. Thus, a large sample size could help ascertain the veracity of this finding.

## 5. Conclusion

Anti‐epo antibody production is not associated with *P. falciparum* infection as well as malarial anaemia. However, anti‐epo antibody production is strongly associated with younger age and higher epo levels.

## Conflicts of Interest

The authors declare no conflicts of interest.

## Funding

No funding was received for this manuscript.

## Data Availability

All relevant data are within the article.
